# Implications of Cross-System Use Among US Veterans With Advanced Kidney Disease in the Era of the MISSION Act: A Qualitative Study of Health Care Records

**DOI:** 10.1001/jamainternmed.2022.1379

**Published:** 2022-05-16

**Authors:** Ann M. O’Hare, Catherine R. Butler, Ryan J. Laundry, Whitney Showalter, Jeffrey Todd-Stenberg, Pam Green, Paul L. Hebert, Virginia Wang, Janelle S. Taylor, Marieke Van Eijk, Kameron L. Matthews, Susan T. Crowley, Evan Carey

**Affiliations:** 1 Hospital and Specialty Medicine Service, VA Puget Sound Health Care System, Seattle, Washington; 2 VA Health Services Research and Development, Seattle-Denver COIN, VA Puget Sound Health Care System, Seattle, Washington; 3 Department of Medicine and Kidney Research Institute, University of Washington, Seattle; 4 Department of Health Services, University of Washington, Seattle; 5 Durham Center of Innovation to Accelerate Discovery and Practice Transformation, Durham Veterans Affairs Health Care System, Durham, North Carolina; 6 Department of Population Health Sciences, Duke University School of Medicine Durham, North Carolina; 7 Department of Medicine, Duke University School of Medicine, Durham, North Carolina; 8 Department of Anthropology, University of Toronto, Ontario, Canada; 9 Department of Anthropology, University of Washington, Seattle; 10 City Block Health, Washington, DC; 11 Department of Medicine, Yale University, New Haven, Connecticut; 12 VA Connecticut Health Care System, West Haven, Connecticut; 13 Center of Innovation for Veteran-Centered and Value-Driven Care, VA Eastern Colorado Health Care System, Denver; 14 Department of Biostatistics and Informatics, Colorado School of Public Health, University of Colorado Anschutz Medical Campus, Denver

## Abstract

**Importance:**

Since 2014, when Congress passed the Veterans Access Choice and Accountability (Choice) Act (replaced in 2018 with the more comprehensive Maintaining Internal Systems and Strengthening Integrated Outside Networks [MISSION] Act), the Department of Veterans Affairs (VA) has been paying for US veterans to receive increasing amounts of care in the private sector (non-VA care or VA community care). However, little is known about the implications of these legislative changes for the VA system.

**Objective:**

To describe the implications for the VA system of recent increases in VA-financed non-VA care.

**Design, Setting, and Participants:**

This qualitative study was a thematic analysis of documentation in the electronic health records (EHRs) of a random sample of US veterans with advanced kidney disease between June 6, 2019, and February 5, 2021.

**Exposures:**

Mentions of community care in participant EHRs.

**Main Outcomes and Measures:**

Dominant themes pertaining to VA-financed non-VA care.

**Results:**

Among 1000 study participants, the mean (SD) age was 73.8 (11.4) years, and 957 participants (95.7%) were male. Three interrelated themes pertaining to VA-financed non-VA care emerged from qualitative analysis of documentation in cohort member EHRs: (1) VA as mothership, which describes extensive care coordination by VA staff members and clinicians to facilitate care outside the VA and the tendency of veterans and their non-VA clinicians to rely on the VA to fill gaps in this care; (2) hidden work of veterans, which describes the efforts of veterans and their family members to navigate the referral process, and to serve as intermediaries between VA and non-VA clinicians; and (3) strain on the VA system, which describes a challenging referral process and the ways in which cross-system care has stretched the traditional roles of VA staff and clinicians and interfered with VA care processes.

**Conclusions and Relevance:**

The findings of this qualitative study describing VA-financed non-VA care for veterans with advanced kidney disease spotlight the substantial challenges of cross-system use and the strain placed on the VA system, VA staff and clinicians, and veterans and their families in recent years. These difficult-to-measure consequences of cross-system care should be considered when budgeting, evaluating, and planning the provision of VA-financed non-VA care in the private sector.

## Key Points

**Question:**

What themes pertaining to cross-system use emerge from review of the Veterans Affairs (VA) health system records of patients with advanced kidney disease?

**Findings:**

In this qualitative study of electronic health records of 1000 US veterans with advanced kidney disease, 3 dominant themes pertaining to VA-financed care outside the VA were identified. Themes described the VA as mothership, the hidden work of veterans, and strain on the VA system.

**Meaning:**

Findings of this qualitative analysis highlight the substantial strain on the VA system, VA staff and clinicians, and veterans and their families of cross-system use.

## Teaser

This qualitative analysis assesses cross-system use of Veterans Affairs (VA)–financed care outside the VA health system from electronic health records of US veterans with advanced kidney disease.

## Introduction

The Department of Veterans Affairs (VA) Health Care System is the largest publicly financed integrated national health care system in the US. While many veterans receive care outside the VA (eg, under Medicare, Medicaid, military and private health insurance)^1^ and the VA has long outsourced some aspects of veteran care to non-VA health systems and clinicians (eg, dialysis, specialized procedures), most VA-financed care for eligible veterans has traditionally been provided within VA facilities.^2^ However, beginning in 2014, amid widespread public concern about prolonged VA wait times,^3,4^ Congress embarked on a series of legislative changes that would increase veteran access to VA-financed health care outside the VA system.^5^

In 2014, Congress passed the Veterans Access Choice and Accountability (Choice) Act, which led to the establishment of a temporary program (the Veterans’ Choice Program) to pay for care outside the VA when this care could not be provided in a timely fashion within the VA.^6^ The Choice Act also authorized allocation of funds to support additional VA staffing and resources to reduce wait times within VA facilities. Preexisting programs and mechanisms for providing VA-financed care outside the VA continued to operate in parallel with the Choice Program. In 2018, the Choice Act was replaced by the more comprehensive Maintaining Internal Systems and Strengthening Integrated Outside Networks (MISSION) Act.^5,7,8,9,10^ Under the MISSION Act, established mechanisms and programs for financing non-VA care (eg, Fee Basis program) were consolidated under the Veterans Community Care Program (VCCP). The MISSION Act was explicit in conferring on the VA responsibility for both financing and coordinating care with non-VA clinicians and health systems to ensure timely scheduling of medical appointments and continuity of care and services.

Eligibility for VA-financed non-VA care under the VCCP varies depending on whether services are available within the VA, whether there is a full-service VA facility within a veterans’ home state, and other considerations such as travel distance, wait time, and clinical need. Access-based eligibility criteria for care under the VCCP are generally more liberal than they were under the 2014 Choice Program, leading to substantial increases in VA-financed non-VA care.^11^ Between 2014 and 2021, VA spending on non-VA care more than doubled from $7.9 billion (approximately 12% of the Veterans Health Administration budget) to $17.6 billion (20% of the Veterans Health Administration budget).^11^ Increasing VA engagement with non-VA clinicians and health systems and responsibility for coordinating much of this care offers a potentially useful model for studying the implications of cross-system use for integrated health systems. Understanding the internal implications of cross-system use for the VA system and enrolled veterans could be relevant to other types of dual system use common in the veteran population (eg, Medicare) and to other health systems.

To date, studies based on interviews with key stakeholders have identified substantial challenges associated with medical record sharing, care fragmentation, timely bill payment, and engagement of non-VA clinicians under the Choice and MISSION Acts.^12,13,14,15,16,17^ To further the understanding of the internal challenges of cross-system use to the VA system and enrolled veterans, we conducted a qualitative analysis of documentation in the VA-wide electronic health record (EHR) pertaining to VA-financed non-VA care in the era of the MISSION Act using an approach that several authors of this study have used to assess other complex care processes.^18,19,20,21^ Analysis of documents and other forms of unobtrusive observation can be helpful in characterizing system-level constructs that might not emerge from interpersonal interviews or fieldwork observations.^22,23^ Because of their traditionally high levels of care complexity and reliance on non-VA clinicians and health systems, our analysis focuses on the medical records of a national sample of US veterans with advanced kidney disease.

## Methods

### Cohort Assembly

We used VA administrative and clinical data to identify a national cohort of veterans alive on June 6, 2019 (starting date for MISSION Act implementation and establishment of the VCCP) with evidence of advanced kidney disease defined as having at least 2 estimated glomerular filtration rate measures below 30 mL/min/1.73 m^2^ at least 3 months apart during the preceding year. We then selected a random sample of 1000 of these veterans for detailed EHR review (Table 1). Information on demographic and clinical characteristics, vital status, and health care use during follow-up were obtained from the VA Corporate Data Warehouse, a comprehensive national repository of information on episodes of care occurring both within (inpatient and outpatient VA encounters) and outside the VA. Specifically, information on non-VA claims was obtained from Patient Integrity Tool data supplemented with information on contract dialysis from the VA Fee Basis files. Race data were reported as recorded in the VA Vital Status file. The project was approved by the institutional review board at the VA Puget Sound Health Care System. Permission to abstract and report deidentified text from cohort member EHRs was approved by the VA Puget Sound Health Care System with a waiver of the requirement for informed patient consent. This study followed the relevant portions of the Consolidated Criteria for Reporting Qualitative Research (COREQ) reporting guideline for qualitative studies.

**Table 1.** Characteristics of Cohort Members With Advanced Kidney Disease

Characteristic	Patients, No. (%) (N = 1000)
Age, mean (SD), y	73.8 (11.4)
Race and ethnicity	
Black or African American	215 (21.5)
White	691 (69.1)
Other (American Indian or Alaskan Native, Asian, Native Hawaiian or other Pacific Islander)	30 (3.0)
Missing	64 (6.4)
Sex	
Male	957 (95.7)
Female	4 (4.3)
Died during follow-up	263 (26.3)
At least 1 claim for VA-financed non-VA care during follow-up, %	607 (60.7)
No. of days with at least 1 VA-financed non-VA care claim during follow-up, median (IQR)	15 (4-94)
At least 1 claim reported for >10% of participants during follow-up	
Anesthesiology	112 (11.2)
Cardiology	164 (16.4)
Dialysis	162 (16.2)
Emergency	266 (26.6)
Home care	191 (19.1)
Imaging	246 (24.6)
Inpatient medicine	275 (27.5)
Laboratory	151 (15.1)
Internal medicine	319 (31.9)
Nephrology	138 (13.8)^a^
Ophthalmology	118 (11.8)
Primary care	196 (19.6)
Surgery	160 (16.0)

Abbreviation: VA, Veterans Affairs.

^a^ Indicates an encounter with a nephrologist not associated with dialysis.

### Data Analysis

We used a Lucene-based search tool (Apache Lucene, version 8.11; Apache Software Foundation) developed by a member of our group (R.J.L.)^18,19,20^ to search text in the VA-wide EHRs of cohort members stored as text integration utilities notes in the VA Corporate Data Warehouse. Because care provided outside the VA under the VCCP (as distinct from other forms of non-VA care not paid for by the VA) is generally referred to within the VA as community care, we searched for mentions of this term in cohort member EHRs between June 6, 2019, and February 5, 2021, after applying a filter to exclude uninformative mentions (eg, boilerplate text).

We analyzed text in the VA-wide EHR identified by the search using inductive content analysis,^24,25,26^ a systematic approach to describing phenomena with the goal of providing new knowledge, fresh insights, and/or identifying opportunities for intervention.^24,25,26^ Using the search tool, 1 coauthor (A.M.O.) reviewed all notes containing mentions identified in our search, abstracting and coding potentially informative text using the constant comparative method.^27,28^ Because veterans can also receive non-VA care that is not financed by the VA, we analyzed only text that clearly pertained to VA-financed non-VA care based on careful review of surrounding text in the EHR (eg, documentation of a referral or authorization). Two other coauthors (C.R.B. and W.S.) independently coded abstracted phrases until reaching thematic saturation. The 3 coauthors (A.M.O., C.R.B., and W.S.) then worked together to develop the thematic schema, identify subthemes, and select exemplar quotations, returning to abstracted phrases and/or the EHR as needed to reconcile any differences in interpretation, clarify meaning, and ensure that emerging themes were grounded in the data.

## Results

The mean (SD) age of the 1000 cohort members was 73.8 (11.4) years. Most (957 [95.7%]) were male, 691 (69.1%) were White, 215 (21.5%) were Black or African American, 30 (3.0%) were from other racial groups (American Indian or Alaskan Native, Asian, Native Hawaiian or other Pacific Islander), and information on race was missing for 64 participants (6.4%). Overall, 607 (60.7%) cohort members had at least 1 active or paid claim for VA-financed non-VA care during follow-up (Table 1). Among the 1000 cohort members, the search identified 583 with at least 1 mention (and a total of 2792 mentions) of the term *community care* in clinical notes between June 6, 2019, and February 5, 2021. Three overlapping and interrelated themes pertaining to VA-financed non-VA care emerged from qualitative analysis of documentation in patients’ VA-wide EHR (Figure). The themes were (1) VA as mothership, which describes extensive care coordination by VA staff members and clinicians to facilitate care outside the VA and the tendency of veterans and their non-VA clinicians to rely on the VA to fill gaps in this care; (2) hidden work of veterans, which describes the efforts of veterans and their family members to navigate the referral process, and to serve as intermediaries between VA and non-VA clinicians; and (3) strain on the VA system, which describes a challenging referral process and the ways in which cross-system care has stretched the traditional roles of VA staff and clinicians and interfered with VA care processes.

**Figure.**
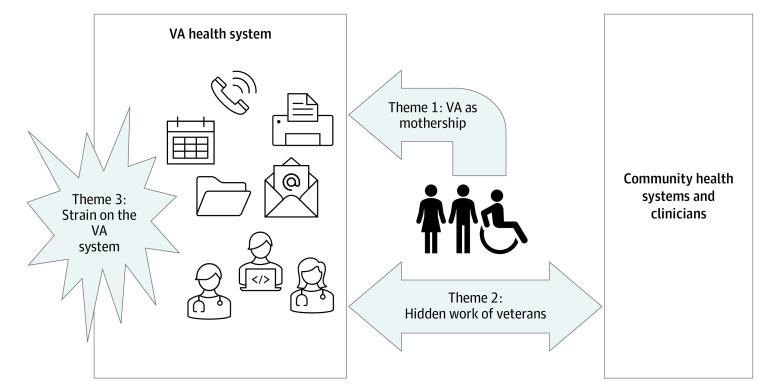
Thematic Schema for Qualitative Assessment Thematic schema based on assessment of documentation in patient electronic health records pertaining to VA-financed non-VA care. VA indicates Veterans Affairs. Illustration was designed by Janelle S. Taylor, PhD.

### Theme 1: VA as Mothership

#### Systematic Care Coordination by Designated VA Staff

Documentation in the EHRs of cohort patients indicated that designated VA staff members (eg, staff within the Offices of Community Care or Access Centers at individual VA medical centers) were engaged in an extensive process of systematic coordination of non-VA care (Table 2). This process involved a range of different tasks including directing requests from non-VA clinicians and health systems to the relevant VA clinicians for authorization, furnishing non-VA clinicians with medical records for referred patients, coordinating between VA and non-VA clinicians to facilitate care, monitoring the care of veterans hospitalized outside the VA and coordinating transfers to the VA when needed, retrieving health records from non-VA health systems, checking on the status and maintaining the momentum of non-VA referrals, and keeping patients’ VA clinicians informed about the care they were receiving outside the VA.

**Table 2.** VA as Mothership Theme and Subthemes

Clinical note title and signatory	Exemplar direct quotation^a^
**Systematic care coordination by designated VA staff**	
VA community care staff RN note	[Secondary authorization request] received from Community Nephrologist requesting referral to Vascular Surgery for Complications of Venous Fistula. Supporting documentation has been indexed and is available for review in Vista Imaging.... If in agreement with referral, please enter a Community Care Vascular Surgery Consult and indicate it is for a [secondary authorization request].
VA community care staff RN note	Contacted Community Care Provider to obtain medical records and provide an update on patient status. Telephone call to provider, spoke to [name of community provider staff member] who confirms care is being received by patient on this consult. Requested treatment sheets and physician's rounding notes. Records request faxed…Will follow as needed.
VA community care staff RN note	Author received call from [name] at [community gastroenterology provider]. She said she received a referral from [patient’s community oncologist]. He wants the veteran to have an endoscopic ultrasound ASAP. [Name] said they have the patient scheduled for a [endoscopic ultrasound].... A [gastroenterology] referral is needed. In addition, the veteran will need to take clear liquids after [time and date] and [nothing by mouth] after midnight [date]. He will need his insulin dose adjusted accordingly so they will need instructions on what dose of insulin to give. Also, the veteran will need to hold the Eliquis 5-7 d prior to the procedure [date]. A note will suffice saying it is permissible to hold the Eliquis starting whatever date is acceptable. The vendor also requested CT [abdomen and pelvis] imaging [compact disc]. Author sent encrypted email to [VA radiology] requesting that they send the imaging [compact disc] via [delivery company] overnight service. Please place [gastroenterology] consult ASAP and request Community Care. Spoke with [name] at [community gastroenterology] regarding plan to hold anticoagulation. Per note below they are requesting 5- to 7-d hold…Patient also has a history of recurrent PE while taking a reduced dose of apixaban. Will await call back from [gastroenterology] to see if they are agreeable to 3-d hold.
**Hidden work of other VA staff and clinicians**	
VA nursing telephone encounter	Patient states he was scheduled on [date] to have x-rays of his knees and an ultrasound of his kidneys requested by his community care Nephrologist. He had transportation problems and was forced to cancel the appointment. His Nephrologist will not see him for his follow-up appointment until this is complete. Requesting to reschedule both exams.
VA nursing telephone encounter note, primary care	Received a notification that [patient name] needs a call back. I called them, I spoke with [patient’s wife], she states that she called last week because [patient] does dialysis in [name of town] and the dialysis doctor is requesting a chest x-ray to [rule out] fluid on the lungs. They are requesting the VA to order it and pay for it in [same town]. Discussed with [VA provider], has entered a Community care referral for x-ray and he has been approved. Called [patient] back to notify. Spoke with [patient’s wife]. Now awaiting on Community Care to select a provider in the network.
VA social work telephone encounter, dialysis	Telephone call received from [name] at [community dialysis provider] stated that [patient] has decided to transfer to [different unit of community dialysis provider] and wants to change modalities to [peritoneal dialysis]. He will need a [peritoneal dialysis] catheter placed…please enter consult for Community Care General Surgery for PD Catheter placement.
VA optometry note, optometrist	Veteran presents with gold weight protruding from skin of upper eyelid …Veteran educated on findings and urgent need for surgical repair.... Scheduled appointment for tomorrow…with [community oculoplastics specialist]…Stressed importance of keeping this appointment, assisted veteran to travel desk for arrangements.
VA psychiatry case management, social worker	Writer provided the contact information [to the Veteran] for the [community care] provider and suggested the veteran attempt to contact them periodically. He is aware the consult could be canceled if he does not respond to the engagement attempts. Writer advised veteran to empty his voicemail.
Physical medicine and rehabilitation outpatient note, physician assistant	Discussed upcoming community care EMG appointment on [date]. Patient is aware of this appointment. Stressed the importance of attending this test in order to help further evaluate his fasciculations and [be] educated on what the test entails so that he will know what to expect. Once test performed, will call facility and request fax of report to have in advance of his follow-up clinic visit.
VA care coordination home telehealth RN note, social work service	I received a call from this veteran reporting he is getting billed for his recent cataract surgery. He reports this is the second bill he has received. I gave him the contact information for the Community Care office and suggested he call them. He agreed. Addendum from Community Care staff: called patient. We will check into situation. Authorization was in place, so we should be able to fix this.
**Veteran reliance on the VA**	
VA primary care RN note	Veteran and spouse presented to the front desk reporting recent discharge from [community medical center]. He was admitted and treated for what the spouse called atrial fibrillation and underwent a cardiac procedure. She is unsure of the exact name but states they'll need a referral to follow-up with cardiologist sometime next week. Veteran and spouse also presented/requested the following prescriptions be filled through the VA.
VA renal social work note	[Patient] contacted renal social worker [date]. Veteran states she has 2 community care medical appointments and requesting writer coordinate her wheelchair roundtrip transportation to both appointments…Writer agreed to enter the travel requests and provide her telephone follow-up once scheduled.
VA emergency department note, emergency medicine physician	[Patient] being seen today in the Emergency Department stating that he signed out of [community hospital] tonight [against medical advice] after being admitted. States he started having some problems with different hospitals…He wants to leave [there to] be admitted here due to his [gastroenterology] doctor knowing him here.
VA primary care nurse practitioner note, home-based primary care	[Patient] informed that per his last visit with his community care pulmonologist in [date], the MD recommended a [follow-up] chest CT to evaluate his lung nodule and to compare with the previous CT that he had done at [name of community hospital]. [Patient] requests that the chest CT be done at [VA facility].
VA primary care RN note	Patient has recently been diagnosed with cancer in the middle of staging and deciding treatment plan… [His niece] also called oncology at [VA medical center] and they can start [treatment] up there while waiting for community care.
VA home-based primary care telephone encounter note, nurse practitioner	[Patient’s] daughter states she will reach out to [primary care provider] if [patient] has future needs for [physical therapy/occupational therapy], changes in condition, or desires hospice care.
VA administrative note, business operations	[Name] with [community hospital] calling to see what can be done to get a new [continuous positive airway pressure (CPAP)] for this veteran as he was in a house fire today…. [Name] states she had called the office of the [community] pulmonologist he sees with no return call. Veteran was supplied the CPAP by the VA. She asked the message be sent to team in case there was something they could do to speed up the process.

Abbreviations: ASAP, as soon as possible; CT, computed tomography; EMG, electromyography; MD, medical doctor; PD, peritoneal dialysis; PE, pulmonary embolism; RN, registered nurse; VA, Veterans Affairs.

^a^ Square brackets denote changes to the original text to protect anonymity or clarify meaning.

#### Hidden Work of Other VA Staff and Clinicians

Other VA staff members and clinicians not officially tasked with coordinating non-VA care were also engaged in less systematic efforts to support this care (Table 2). This support included helping veterans to access services both within and outside the VA that had been recommended by their non-VA clinicians, encouraging veterans to keep non-VA care appointments, and helping to set up travel to non-VA appointments. Veterans Affairs staff and clinicians also sometimes coached veterans on how to interact with non-VA contractors and clinicians. Medical teams routinely relayed information, questions, and concerns (eg, unexpected medical bills) about non-VA care to VA Community Care staff members.

#### Veteran Reliance on VA

The work of VA staff and clinicians to support VA-financed non-VA care was in part prompted by the tendency of veterans to turn to the VA for assistance with referrals for non-VA care and filling administrative and clinical gaps in the care they were receiving (or wished to receive) outside the VA (Table 2). There were also instances of veterans seeking the care of trusted VA clinicians to supplement or replace VA-financed care they were receiving outside the VA. Incidentally, we found examples of veterans turning to the VA for bridging care while waiting for an appointment with a non-VA clinician, and of VA clinicians encouraging patients to return to the VA if specific services were needed. Documentation in veterans’ EHRs suggested that non-VA clinicians also sometimes helped or encouraged veterans to access VA services and resources to fill gaps in the care they were receiving outside the VA.

### Theme 2: Hidden Work of Veterans

#### Navigating the Referral Process

Despite the efforts of VA staff and clinicians, procurement of VA-financed non-VA care demanded substantial time and effort on the part of veterans and their families (Table 3). Documentation in the VA EHR suggested that veterans did not always find it easy to engage with the VCCP referral process in ways expected of them. We found examples of referrals that had been stalled or canceled due to the veteran not answering their phone or not responding to (or being confused by) calls they had received about their non-VA care. Veterans were expected to be proactive in initiating and maintaining the momentum of referrals, and many were. However, they also struggled with the referral process and had difficulty accessing needed care, which could be time-consuming and anxiety-provoking. There were also examples of veterans receiving bills for care they had received outside the VA, either in error or when care had not been preauthorized by the VA. Documentation in the EHR suggested that the reality or prospect of being billed for non-VA services weighed heavily on veterans and their families.

**Table 3.** Hidden Work of Veterans Theme and Subthemes

Clinical note title and signatory	Exemplar direct quotation^a^
**Navigating the referral process**	
VA psychiatry case management note, social worker	[Veteran] claims he never spoke with anyone from Office of Community Care; however, writer explained they have documented their attempts, which includes mailing letter. Writer provided the contact information for the Office of Community Care and offered to call with him, but veteran declined after saying, “I'll get to it Monday. It's too late in the day.” He is aware the consult will be canceled if he does not contact the Office of Community Care.
VA primary care administrative note, medical support assistant	Patient presented to clinic to inquire about community care nephrology consult. Consult appears to still be in process of approval. Patient is anxious about seeing a provider regarding his renal function.
VA primary care secure messaging, LVN	I'm very frustrated. My husband is in pain. He needs to get his hyaluronate bilateral knee injection…I requested a referral to get the injections done outside the VA. The person I talked to could not help me. Someone PLEASE get my husband a referral through Community Care to get these injections done.
VA primary care RN telephone note	Veteran reported that he only received 8 treatments and was informed by the contractor that he needs a new referral…Advised veteran to contact [Community Care]…Veteran voiced that he received a written statement for a number of sessions from the contractor and it's different than stated on the request. Veteran voiced “with different info, I feel like I am left alone.”
VA attending note, nephrology	[Veteran] understood he was referred for transplant eval[uation] under VA auth[orization]. He stated [community hospital] providers then recommended imaging, biopsy, etc. for suspected cancer that was discovered during transplant eval[uation]. He proceeded with scheduling those recommended procedures at [community hospital] under the assumption the VA was paying for the care. Social worker explained that since the VA is not an insurance provider, bills for community care are only paid if the VA gives a prior authorization…Veteran was upset and feels [community hospital] did him a disservice by not referring him back to the VA.
VA social work note	The patient's wife called and explained that his nephrology bill from [month] has been referred to a collections agency. The patient has authorization for nephrology [community care], explained that [we] would enter a notation regarding the authorization, and that the patient should not receive further bills…Provided empathic listening and validation.
VA administrative note, RN	Veteran [left voice message] stating that he is in the hospital getting ready to have “kidney surgery.” Veteran asking for status of consult placed in October for Urology… Veteran concerned about Urology consult not being completed and if he needs a consult for surgery tomorrow.
VA primary care RN note	Patient was instructed he needs to go to the [emergency department]. He states, “I don't have insurance, will the VA pay?”…Explained I cannot guarantee payment, but I will let [VA medical center] know we sent you.
**Veteran as intermediary**	
Primary care secure messaging, RN	I just completed an appointment with [name of community nephrologist]. I recently had full lab and ultrasound done. She said she is forwarding a note to you that I need to see a urologist ASAP.
VA primary care RN telephone encounter	Veteran dropped off 2 [prescriptions] at the Southeast VA Clinic from [community provider]. [Prescription] dated [date]: “Remove Hemo Dialysis Catheter” and “Cholecalciferol 1000 units Daily #90 with 3 refills” written by [community provider].
VA primary care telephone RN note	Veteran is scheduled for surgery tomorrow through Community Care Dermatology. Dermatology office has not received authorization papers…Wife requesting for [VA primary care team] to call her when paperwork is faxed to the doctor’s office.
MyHealtheVet Dialog note (VA’s electronic messaging portal), community care RN	My wife has made several [attempts] to contact the dentist's office in [city]. The dentist's office needs VA approval before they will see me about a piece of bone or tooth that is sticking out of my gum. THEY PULLED MY TEETH BACK IN [three months prior]. PLEASE HELP it is hurting me.
VA cardiology procedure note, medical technologist	[Patient] left a voicemail for author stating he saw the private cardiologist at [name of medical center], had his device checked, and is now scheduled for generator change-out procedure [time and date]…This morning [patient] states he is scheduled for pacemaker interrogation with private cardiologist [time and date] with [patient] noting that after that he will be “turned loose.”
VA primary care nursing note	Contacted veteran for status [follow-up], veteran stated “I'm doing ok,” reports [community provider] admitted patient “couple of days ago, took my blood; you should have my results in my chart,” no results noted in chart. [Nurse] will contact [community provider] for lab results.
VA primary care outpatient note, physician	Consultation to [dermatology] was placed. He is eligible for community care, and that option was chosen. If he does not hear from them in 10-14 d, he is to let us know.

Abbreviations: ASAP, as soon as possible; LVN, licensed vocational nurse; RN, registered nurse; VA, Veterans Affairs.

^a^ Square brackets denote changes to the original text to protect anonymity or clarify meaning.

#### Serving as Intermediaries

The records and treatment recommendations of non-VA clinicians were frequently not available to VA clinicians in a timely fashion, requiring that veterans (and/or their family members) serve as informants and messengers between their VA and non-VA clinicians (Table 3). We found examples of veterans requesting initiation, continuation, and/or expansion of coverage for particular services at the behest of their non-VA clinicians and conveying messages about treatment recommendations across systems. Veterans and/or their family members also provided VA clinicians with important contextual information about the care they were receiving (or had received) outside the VA.

### Theme 3: Strain on the VA System

#### Challenging the Referral Process

There were multiple references in medical documentation to the time-consuming and inefficient nature of the community care referral process (Table 4). By design, VA referrals for care outside the VA were time limited, the scope of services covered by each referral was prespecified, and referrals were intentionally canceled when veterans did not respond to telephone calls. Requests for continuation of services had to be authorized by VA clinicians as did any changes to, or expansion of, authorized services, and canceled consultations had to be resubmitted. Veterans Affairs staff and clinicians appeared to have limited control and understanding of the referral process after submitting the consultation and were often uncertain about the status of particular referrals. Clinicians were also sometimes unsure who to turn to for support and assistance with tracking referrals and confirming that needed non-VA care had in fact been (or would be) provided.

**Table 4.** Strain on the VA System Theme and Subthemes

Clinical note title and signatory	Exemplar direct quotation^a^
**Challenging referral process**	
VA community care staff RN note	[Community physician] is requesting the following additional services…Please review and if continued care is clinically indicated and cannot be provided [by VA primary care], please enter a new Community Care Cardiology consult to continue care for this Veteran…Thank you!
VA community care RN staff note	Clinical Note: Community Care Approved, Program…may discontinue if Veteran cancels/no-shows twice or fails to respond to mandated scheduling effort.
VA primary care note, nurse practitioner	Seen by a VA neurologist as well as non-VA neurosurgeon.... The VA neurologist put in for community care for physical therapy which appears has been discontinued. He also put the wrong order…That was also discontinued. I requested…VA physical therapy consult.
VA primary care telephone encounter note, nurse practitioner	I truthfully cannot figure out if he is authorized now or not for [community provider] surgery. I asked him to come for his posthospital visit tomorrow but first attempt to contact [community provider]. If need[s] be I will send another consult to neurosurgery.
VA primary care secure messaging, LPN	By any chance did Dr [name of VA physician] submit a request to get my colon checked by a civilian doctor? Hi there [patient name], Yes. Dr [name] submitted a consult on [date] for a local colonoscopy. These seem to go through slowly (obviously). You can always call Community Care at [phone number]…to see if they can tell you where the authorization is in the process. I hope you have a wonderful Thanksgiving!
VA primary care telephone note, nurse practitioner	[Patient] has been getting bills from [community hospital] for his cataract surgery that he had done through community. I advised that he should call the Office of Community Care about this. I was also wondering if this was something social work could help with.
Nephrology telephone note, RN	Community Care consult for home phlebotomy to optimize veteran's care, adhere to COVID-19 social distancing recommendations/precautions–hopefully this will be approved.
**Stretching VA staff roles**	
VA social work risk assessment screening note, medical service	[Patient’s wife] stated that she would like to resume Respite Care services…[social worker] informed [patient’s wife] that [patient’s primary care provider] will be alerted to submit the consult.
VA renal social work note	[Community provider] nephrology billing department contacted this worker to discuss issues with receiving payments for nephrology services.... [Social worker] reviewed consult for nephrology …. Consulted with Community Care office to work out solution. [Social work] will provide proper authorization format, if necessary, to [community provider] so that bills can be resubmitted.
VA renal social work note	Home health [physical therapist assistant] also states veteran's glucose levels are not controlled and veteran needs to be seen by [primary care provider]. Informed [physical therapist assistant] that writer is able to request the early pickup for [date] and writer will inform veteran's nephrologist…of the above.
VA primary care social work telephone encounter	Call from [patient] who stated that he spoke with…business [department] with [dialysis provider]. He stated that [name] told him to have his nephrology social worker call her with the VA authorization number and dates of service. This writer explained that this writer is not a nephrology social worker but a primary care social worker but would assist him.
VA primary care RN note	The veteran was informed to follow up with his Cardiologist about the above medication and to get them from his Cardiologist, due to changes in dosage and adjustment that may be needed. They were made aware that [VA physician] is his Primary Doctor and the above medication needs to be managed by his Cardiologist.
Renal social work note	Veteran states “I need a fistulogram because my arm is tingling/going numb.” Writer informed veteran of the vascular access referral process to include veteran's community care dialysis unit faxing an access referral form and accompanying medical records to [VA medical center] access nurse coordinator. Veteran verbalized understanding of the above and requested writer contact [community] dialysis unit regarding her request. Writer agreed and informed veteran once referral is received [VA] interventional radiology department will contact her for scheduling.
**Interaction with VA care processes**	
VA physical medicine and rehabilitation, physician	I rescheduled [patient’s] appointment to [date and time] due to his conflicting community care neurology appointment at [community provider] on [same date].
VA telephone encounter note, gastroenterology nurse practitioner	Explained about the risks of high creatinine levels and requested the patient to come to the [emergency department] for further management. Patient declined at this time, since he has an upcoming community care Doppler imaging scheduled on Monday [date] and does not want to be canceled for no reason.
VA cardiology consult, cardiology nurse practitioner	He was planned to be followed in our heart failure clinic after prior admissions, but he chose to have community care. Cardiology at the VA was consulted again after difficulty getting him timely community care appointments.
VA renal social work note	[Patient] contacted social worker regarding her special mode transportation…Veteran states her [date] community care dental appointment...was canceled and rescheduled for [date]. Veteran requesting writer reschedule her roundtrip transportation for the new appointment date. Writer informed veteran that she would enter the request; however, could not promise the request could be processed since it was requested less than 24 h prior to date.
Primary care telephone note, physician	Please let patient know that recent labs raised the following issues: His hemoglobin is trending down. His creatinine improved, a bit. I see that Community Care is helping him get an appt with an outside nephrologist. Please ask him if a date for the visit has been set yet. Once [we] know that date, we can make a plan.
VA community care staff RN note	Amended visit note with [community nephrology provider] additional recommendation for oncology referral for M-spike on serum electrophoresis. Lab result requested, vendor is unable to locate the lab results, alerting care team to review amended visit note and repeat serum electrophoresis for clinical assessment.
VA nephrology telephone note, nurse practitioner	Have attempted to optimize close lab monitoring including use of community care home lab monitoring; however this system has not been consistently successful. Veteran reports this last request, he did receive a call from VA Community Care, and also a call from the home lab phlebotomist; however no one showed up to his home.
VA hematology and oncology note, medicine resident	[Return to clinic] in 4 weeks with labs if community care is not yet setup.

Abbreviations: LPN, licensed practical nurse; RN, registered nurse; VA, Veterans Affairs.

^a^ Square brackets denote changes to the original text to protect anonymity or clarify meaning.

#### Stretching Traditional VA Staff Roles

The high level of VA clinician oversight required by the referral process meant that VA Community Care and other support staff routinely routed referral requests to physicians for approval, bureaucratizing their clinical role (Table 4). Efforts to accommodate the needs of veterans receiving care outside the VA also stretched the traditional roles of other VA clinical staff members. This was especially true for internal VA clinical social workers who often served as the point of contact for veterans and their non-VA clinicians seeking assistance with the referral process. Because those seeking such assistance were usually not familiar with the inner workings of the VA system or the VCCP, these requests were often misdirected, and VA staff and clinicians were not always accommodating.

#### Interaction With VA Care Processes

Referring veterans to outside clinicians and health systems could also interact and conflict with VA care processes (Table 4). We found examples of VA clinicians rearranging VA appointments to accommodate veteran appointments outside the VA. Changes or delays in the provision of non-VA care limited the ability of the VA to help coordinate or otherwise support this care (eg, arranging transportation). Lack of information about care delivered outside the VA or the status of particular referrals led to duplication of services and increased the work of VA clinicians while limiting the quality and timeliness of the care they were able to provide. Clinicians also routinely made contingency plans (eg, placeholder appointments) to accommodate uncertainty about whether and when non-VA services would be initiated.

## Discussion

This qualitative study of documentation in the VA-wide EHR among a national random sample of 1000 US veterans with advanced kidney disease offers an internal window on the challenges of coordinating care across health systems. Specifically, our findings spotlight the substantial work of VA staff and clinicians and veterans and their families to arrange and coordinate VA-financed care outside the VA, and the strain that this can place on VA care processes. Although some of our findings pertain specifically to the VCCP in the era of the MISSION Act, most speak to the broader challenges of caring for patients receiving care in different health systems and thus bear relevance to other kinds of cross-system use among VA users (eg, VA-Medicare dual use) and to other health systems.

Since the VA waitlist crisis of 2014, there have been major changes to the types and volume of veteran care referred to non-VA clinicians and health systems. According to a recent Congressional Budget Office report, the number of veterans authorized to receive VA-financed care outside the VA almost doubled between 2014 and 2020 from 1.3 to 2.3 million.^11^ In 2021 alone, the VA made more than 6 million referrals for non-VA care for eligible veterans.^29^ It is thus perhaps not surprising that, similar to earlier interview studies of VA cross-system use under the Veterans’ Choice Program,^12,13,14,15,16^ our results echo familiar refrains about health care fragmentation and complexity including surprise medical billing,^30^ the work involved in being a patient,^31,32,33,34^ and the invisible work of family members to support patient care.^31,32,35^ The official responsibility of the VA for coordinating VA-financed care in the community along with several of the dynamics that emerged from our analysis of the VA EHR (ie, the mothership theme outlining the work of designated VA Community Care staff to coordinate non-VA care under the VCCP, the informal work of nondesignated VA staff and clinicians to support this care, and the tendency of veterans to turn to the VA to fill gaps in non-VA care) may well have increased the visibility of these somewhat hidden and difficult-to-measure consequences of cross-system use. Together with earlier interview-based studies,^12,13,14,15,16,36,37,38^ our results highlight the importance of finding ways to account for the many intangible consequences of cross-system use when budgeting, evaluating, and planning the delivery of VA-financed care outside the VA.

Although many of our findings speak to the general challenges of cross-system use, they also have specific relevance to the VA health care system. In the wake of the Choice and MISSION Acts, the VA has been required to interact on an unprecedented scale with private health systems, many of which do not share its programmatic strengths or mission and culture of providing lifelong care to the veteran population.^2,39^ It is thus perhaps not surprising that our description of the work of VA staff and clinicians to support veterans to receive VA-financed care outside the VA is consistent with the work of Lamphere^40^ on Medicaid reform in New Mexico in the late 1990s, which described the substantial hidden work that public safety net institutions and clinicians undertook to try to smooth client interactions with other health systems when their care was shifted to the private sector.^40^ Our findings also show consistency with the work of Van Eijk^41^ describing the mission-based approach of staff in an inner-city transgender clinic to supporting client interactions with insurance companies and other elements of the health system. Although the Choice and MISSION Acts were intended to improve the timeliness of veteran care by increasing access to non-VA clinicians, it is presently unclear whether the substantial VA investment in non-VA care in recent years has accomplished this goal,^42,43^ at least in sufficient measure to justify the increased demands placed on the VA system, VA staff and clinicians, and veterans and their families.

### Limitations

While we describe dominant themes pertaining to the provision of VA-financed non-VA care as documented in the VA-wide EHR, our findings may not speak to the overall strengths and limitations of the VCCP for the following reasons: (1) the VA EHR does not directly or completely capture the experiences or perspectives of veterans, family members, or VA staff and clinicians; (2) we did not evaluate veteran interactions with private health systems and clinicians or capture the experiences of non-VA staff and clinicians; (3) we may not have adequately described the care of veterans who interacted infrequently with the VA system during the observation period; (4) our study focused on veterans with advanced kidney disease because of their high levels of care complexity and cross-system use, which may limit the transferability of our findings to veterans with other health conditions; (5) we described the early rollout of the VCCP and thus our results do not provide meaningful information on program evolution; and (6) our analyses were not designed to distinguish between particular non-VA contractors, clinicians, or types of care and thus may not address important differences across these domains.

## Conclusions

In this qualitative study of documentation in the VA-wide EHRs of US veterans with advanced kidney disease pertaining to VA-financed care outside the VA, some of the challenges of cross-system use and specifically the strain this can place on health systems, clinical personnel, patients, and families were highlighted. These difficult-to-measure consequences associated with cross-system use warrant consideration when budgeting, evaluating, and planning for the provision of VA-financed non-VA care for the US veteran population.
